# Evidence that a tax on sugar sweetened beverages reduces the obesity rate: a meta-analysis

**DOI:** 10.1186/1471-2458-13-1072

**Published:** 2013-11-13

**Authors:** Maria A Cabrera Escobar, J Lennert Veerman, Stephen M Tollman, Melanie Y Bertram, Karen J Hofman

**Affiliations:** 1 PRICELESS SA (Priority Cost Effective Lessons in System Strengthening South Africa), Johannesburg, South Africa; 2 School of Population Health, The University of Queensland, Brisbane, Australia; 3 Wits/Medical Research Council Rural Health and Health Transitions Unit, School of Public Health, Faculty of Health Sciences, University of Witwatersrand, Johannesburg, South Africa

**Keywords:** Obesity, Fiscal policy, Tax, Non-communicable diseases (NCDs), High income countries, Middle income countries, Sugar Sweetened Beverages (SSBs), Elasticity, Demand, Price

## Abstract

**Background:**

Excess intake of sugar sweetened beverages (SSBs) has been shown to result in weight gain. To address the growing epidemic of obesity, one option is to combine programmes that target individual behaviour change with a fiscal policy such as excise tax on SSBs. This study evaluates the literature on SSB taxes or price increases, and their potential impact on consumption levels, obesity, overweight and body mass index (BMI). The possibility of switching to alternative drinks is also considered.

**Methods:**

The following databases were used: Pubmed/Medline, The Cochrane Database of Systematic Reviews, Google Scholar, Econlit, National Bureau of Economics Research (NBER), Research Papers in Economics (RePEc). Articles published between January 2000 and January 2013, which reported changes in diet or BMI, overweight and/or obesity due to a tax on, or price change of, SSBs were included.

**Results:**

Nine articles met the criteria for the meta-analysis. Six were from the USA and one each from Mexico, Brazil and France. All showed negative own-price elasticity, which means that higher prices are associated with a lower demand for SSBs. Pooled own price-elasticity was -1.299 (95% CI: -1.089 - -1.509). Four articles reported cross-price elasticities, three from the USA and one from Mexico; higher prices for SSBs were associated with an increased demand for alternative beverages such as fruit juice (0.388, 95% CI: 0.009 – 0.767) and milk (0.129, 95% CI: -0.085 – 0.342), and a reduced demand for diet drinks (-0.423, 95% CI: -0.628 - -1.219). Six articles from the USA showed that a higher price could also lead to a decrease in BMI, and decrease the prevalence of overweight and obesity.

**Conclusions:**

Taxing SSBs may reduce obesity. Future research should estimate price elasticities in low- and middle-income countries and identify potential health gains and the wider impact on jobs, monetary savings to the health sector, implementation costs and government revenue. Context-specific cost-effectiveness studies would allow policy makers to weigh these factors.

## Background

Obesity is a global epidemic and is a major risk factor for the growing burden of non-communicable diseases (NCDs) including heart diseases, diabetes and some cancers [1,2]. Although previously considered a problem of high income countries (HICs), NCDs are now having a major impact on the economy of low and middle income countries (LMICs) [3]. In the past three decades there have been considerable changes in lifestyle around the world, helped along by globalisation of the food supply and urbanisation [4]. These changes affect diet and decrease levels of physical activity, thereby increasing body mass index (BMI) [5]. The Global Burden of Disease (GBD) study (2010) shows an increased share of non-communicable diseases in adults over the period from 1990 to 2010, both globally and in each region [6]. Dietary risk factors (including low intake of fruits, vegetables, whole grains, nuts and seeds, and omega-3 fatty acids) and physical inactivity are estimated to be responsible for ten percent of the global health loss, expressed as disability-adjusted life years [6].

Middle income countries, in particular, face many challenges given far-reaching epidemiological and demographic transitions underway [7,8]. For example, South Africa reported the highest prevalence of people classified as overweight or obese (29% of men and 56% of women) of all countries in Africa [9]. Data from the National Burden of Disease Study in South Africa show that NCDs were responsible for 28% of the disease burden in 2004, which is similar in magnitude to the HIV/AIDS burden [10].

Worldwide, despite lifestyle change programmes to prevent obesity, this epidemic is growing. Interest is mounting in developing combined approaches to address individual behaviour change together with population-oriented fiscal policies such as tax and subsidies to encourage healthier food consumption patterns. For example, in Hungary, a “fat tax” is part of a fiscal policy to address the obesity epidemic [11]. Several states in the USA have also introduced an excise tax on sugar sweetened beverages (SSBs), the original intention being to raise revenue, but this is now considered as anti-obesity policy [12]. While many studies to date come from HICs, there is limited information in the published literature regarding legislative and fiscal changes policy in LMICs.

Various categories of food products have been recommended for policy action to improve health. These include processed food (high in salt, sugar and fat), high energy density food (energy density refers to the amount of energy in a given weight of food in kcal/g or kJ/g) [13], fast food, food containing saturated fat, junk food and soft drinks [14]. In South Africa, legislation has now been passed to regulate salt in processed food [15,16]. With respect to obesity, an effective starting point to diminish unhealthy food consumption might be through taxing of SSBs [17]. The application of an excise tax on these products is an attractive option because, while SSBs are a significant contributors to the energy intake in many populations (for example, they account for about 7% of all calories consumed in the US [18]; whereas for US children and adolescents this is 11% [19]) they provide little or no nutritional value [20-23]. Further, SSBs are marketed aggressively [24] and are easily accessible to all age groups through vending machines, restaurants, schools, cafeterias and shops, as well as at home [25,26]. A recent review concludes that “the cumulative evidence from observational studies and experimental trials is sufficient to conclude that regular consumption of SSBs causes excess weight gain” [19].

The link between intake of SSBs and obesity-related health outcomes is well established [27-29], as is the link between the intake of SSBs and conditions such as osteoporosis [30] and dental caries [28]. From an early age this poses a risk of low nutrient intake because children tend to substitute SSBs for healthier drinks [28,31]. A range of clinical trials and cohort studies provide evidence for a causal association between the intake of SSBs and obesity [32]. It is possible that a subgroup of individuals with a greater genetic predisposition may be more susceptible to obesity induced by SSBs [29].

Interventions outside the health care system can have a significant impact on a nation’s health, as recognised in WHO’s 'health-in-all-policies’ framework [33]. Small changes in diet for many individuals can translate into large population health gains at relatively low cost [34] and government finance departments in particular can improve population health by establishing incentives and disincentives to drive change throughout the food system, including consumer purchases [35]. If government departments that do not have health as their primary responsibility are to consider health-promoting interventions, evidence on the effectiveness of those options is needed [36].

This review evaluates the published evidence for SSB taxes or price increases, and their potential impact on consumption levels and effects on obesity, overweight and BMI. The possibility of switching to alternative drinks is also considered. Two previous studies have reported pooled estimates of price elasticities in the consumption of SSBs, but both were restricted to data from the US [37,38]. The present study will also include non-US studies, and so contribute to the evidence base on the contribution that SSB taxation can make to improving diets and health around the world.

## Methods

A systematic literature review was conducted, including original research articles, working papers, and editorials related to SSBs. Articles published between January 2000 and January 2013 were selected.

Many terms are used to describe SSBs. These include soft drinks, sugary drinks, non-alcoholic drinks, soda, sweet drinks, beverage, fruit drinks, sport drinks, cold drinks and carbonated SSBs. Other beverages including full cream milk, low-fat milk, skim milk, water, tea and coffee were excluded. These drinks may contain some nutritional value and none of them contain sugar added prior to packaging, so their relationship with obesity is not as direct it is for SSBs.

The inclusion criteria were articles in English from any country, with original evidence on the quantitative impact of SSB price changes on the consumption of SSBs, consumption of other drinks, or weight loss, obesity or BMI. For the meta-analysis, we excluded articles that did not report standard error or confidence interval on the own-price elasticity and/or cross-price elasticity. Articles that did not clearly define the type of SSBs were also excluded.

**Figure 1:**
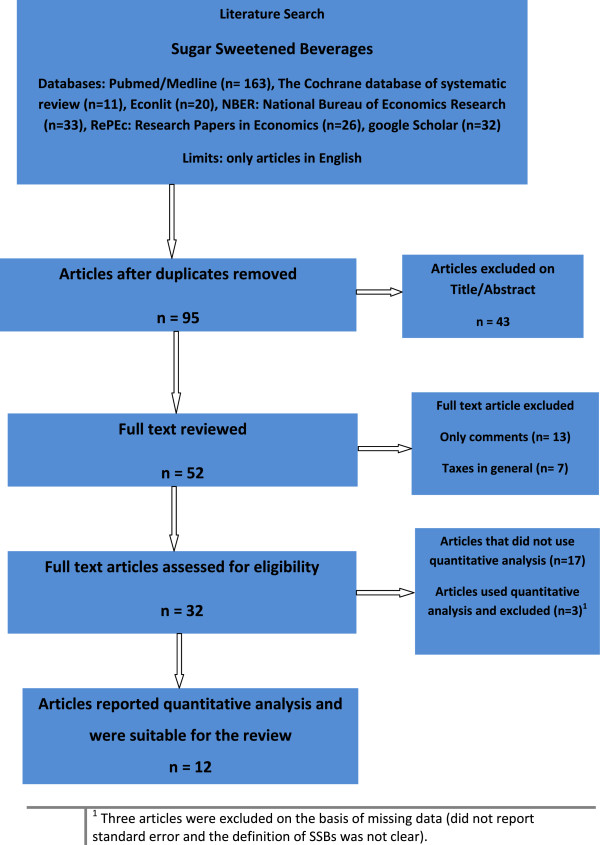
Literature search.

Figure 1 shows the literature search according to the following key words and databases:

Key words searched were: “nutrition AND policy AND tax”, “legislation AND obesity”, “tax food”, “elasticity of demand”, “policy AND “soft drink””, “policy AND “fast food””, “food AND prices”, “elasticity AND nutrition”, and “fat tax”.

Databases used were: Pubmed/Medline, The Cochrane database of systematic reviews, Google Scholar, EconLit (AEA), National Bureau of Economics Research (NBER), and Research Papers in Economics (RePEc).

The first author carried out the search, applied the inclusion criteria and extracted the data, in frequent consultation with a second researcher (JLV). We used an MS Excel-based data extraction form that was piloted on a subset of the data and subsequently revised. The following quantitative results were extracted: own-price elasticity (percentage change in quantity demanded and standard error), cross price elasticity (percentage change in quantity demanded and standard error) (see Table 1 definitions) [39,40], or impact on obesity (percentage points and standard error), overweight (percentage points and standard error) or BMI (kg/m2 and standard error), due to a tax or price change on SSBs. If the data was presented in other metrics values, this was changed to the metrics value of interest using the BMI and cross-price elasticity formula. If the articles lacked certain data or if the analyses were unclear, the authors were contacted for additional information or clarification.

**Table 1:** Definition of price elasticity

**Own-price elasticity**	**Cross-price elasticity**
To estimate the impact of taxes on specific foods, it is important to know just how responsive quantity demanded is to change in price. “Own-price elasticity” is an index that expresses this responsiveness. It is the ratio of the percentage change in quantity demanded to the percentage change in price. This should be negative, because the demand for certain products normally decreases as its price increases. If the own-price elasticity is greater than the absolute value of 1, the demand is called 'elastic’. If it is less than 1, demand is inelastic [39,40].	A related concept is’cross-price elasticity’, which measures the change in the quantity demanded of one good in response to a change in the price of another good. It can be either positive or negative. Positive cross-price elasticity indicates that an increase in the price of X causes the demand for Y to rise. This implies that the goods are *substitutes*. A negative cross-price elasticity indicates that an increase in the price of X causes a decrease in the demand for Y, which implies that the goods are complements [39,40]

Meta-XL, a tool for meta-analysis in Microsoft Excel, was used to synthesize results for own- and cross-price elasticities using the random effects model (http://www.epigear.com). A funnel plot test was performed to check for possible bias in the own-price elasticities incorporated in the meta-analysis. The studies reporting weight outcomes were too heterogeneous to be pooled.

## Results

Figure 1 shows the results of the literature search. First, of 95 articles related to SSBs, 43 were excluded as the title or abstract indicated that the article was not related to fiscal or legislative policies. Second, out of the 52 full text articles reviewed, 20 were excluded because they only commented on SSBs (without mention of any policy) or discussed taxes in general (not directly related with SSBs).

Of the remaining 32 articles, 15 presented quantitative data and the remaining 17 reported qualitative data (Figure 1). Three further articles were excluded on the basis of missing data (did not report standard error and the definition of SSBs was not clear). These three studies were performed in Germany [41] and two in the USA [42,43].

The twelve articles that qualified for our analysis reported own-price elasticity, cross price elasticity, or impact on obesity, overweight or BMI. Nine articles met the criteria for the meta-analysis (Table 2). The remaining three articles reported only the impact of SSB price on BMI, overweight and/or obesity. All primary data consisted of either cross sectional or longitudinal studies. There were no intervention studies. The studies were from four countries: USA, France, Mexico and Brazil. All were published between 2008 and 2013.

**Table 2:** Characteristics of the 12 articles in the meta- analysis and the impact on obesity, overweight and BMI

**Author**	**Population (n)**	**Dataset**	**Type of SSBs**	**Independent variable**
Barquera S, et al. [44], Mexico	1) Adolescents (n = 416)	1) Mexican Nutrition Survey Adolescents, 1999	Soda	Price
	Adults (n = 2180)	2) Mexican Health and Nutrition Survey, 2006		
	2) Adolescents (n = 7464)	3) The Mexican household income and expenditure surveys, 1989, 1998 and 2006	Sweet drinks	
	Adults (n = 21 113)			
	3) Household (n = 12 000 to 20 000)			
Bonnet C, et al. [45], France	Household (n = 19 000)	Consumer panel data	Soft drink	Price
		2003-2005		
Claro RS, et al. [46], Brazil	All ages	Household food consumption data	SSB	Price
	(n = 48470)	2002-2003		
Dharmasena S, et al. [42], USA	Household	Nielsen Homescan Panel	Non-alcoholic beverage	Tax
		1998 to 2003		
Finkelstein EA, et al. [18], USA	Household	Nielsen Homescan panel	Carbonated SSBs	Price
	(n = 384 252)	2006	All SSBs	
Finkelstein EA, et al. [47] USA	Adults and children	Nielsen Homescan panel	SSBs	Price
		2006		
Fletcher JM, et al. [12], USA	Ages >18	Behavioral Risk Factor Surveillance System 1990 – 2006	Soft drink	Tax
Fletcher JM, et al. [48], USA	Ages 3 to 18 (n = 34 000)	NHANES 1989 and 2006	Soft drinks	Tax
Han E and Powell LM. [23], USA	Follow-up after high school graduation	Monitoring the Future Surveys	Soft drink	Price
	(n = 2 400)	1992-2003		
Lin BH, et al. [49], USA	Household (n = 22 750)	Nielsen National Consumer Panel	Sugary drinks	Price
	Children 2–19 (n = 7291 Children)	1998-2007	Diet drinks	
	Adults 20 and older (n = 8 322)	National Health and Nutrition Examination Survey	Juices	
		2003-2006		
Powell LM, et al. [21], USA	Students from 8th - 10th grade	Monitoring the future Survey combined with state-level tax data and local area contextual measure 1997-2006	Vending machine soda	Tax
	12 000 – 15 000 Students			
	from 12th grade (n = 30 000)			
Smith TA, et al. [50] USA		Nielsen Homescan panelists	Caloric sweetened beverages	Price
		1998 – 2007		

**Figure 2:**
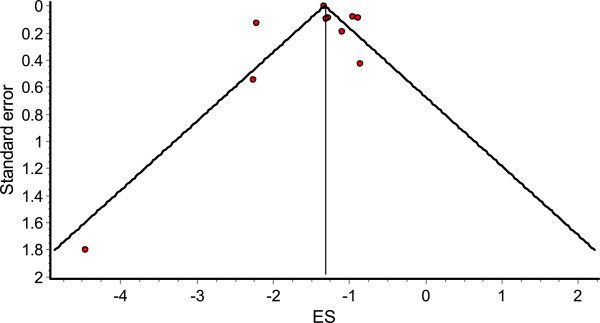
Funnel plot of studies with own-price elasticity of SSBs as outcome.

Table 3 shows ten estimates of own-price elasticity reported in nine of the studies: six from the USA [18,47-51] and one each from Mexico [44], Brazil [46] and France [45]. Of the studies performed in the USA, two used tax data and four price data, while the studies performed in Mexico, Brazil and France used price data. All the results show negative elasticity, which means that an increase in price was associated with a decrease in the demand for SSBs. Of the studies done in middle income countries, the one in Brazil showed an elasticity of -0.85 [46] and the one in Mexico, -1.085 [44]. The results from the meta-analysis show that the pooled elasticity estimate is -1.30 (95% CI: -1.089 – -1.509). The funnel plot (Figure 2) for the own-price elasticities in the meta-analysis was roughly symmetric, which provides no indication of publication bias.

**Table 3:** Own and cross price elasticity of SSBs

**Author/year/country**	**Own-price elasticity**		**Cross-price elasticity**					
	**Estimated**	**SE**	**Fruit juice**	**SE**	**Whole milk**	**SE**	**Diet drink**	**SE**
**Barquera S, et al. (2008)**[44]**, Mexico**	-1.085	0.195	-0.016	0.003	0.052	0.011		
**Bonnet C, et al. (2011)**[45]**, France**^**1**^	-2.206	0.133						
**Claro RS, et al. (2012)**[46]**, Brazil**^**2**^	-0.85	0.434						
**Dharmasena S, et al. (2012)**[42]**, USA**^**3**^	-2.255	0.550						
**Finkelstein EA, et al. (2010)**[47]**, USA**	-0.870	0.090						
**Finkelstein EA, et al. (2013)**[18]**, USA**	-1.320	0.005						
**Fletcher JM, et al. (2010b)**[48]**, USA**^**4**^	-4.445	1.806	1.857	2.332	7.67	2.156		
**Lin BH, et al. (2011)**[49]**, USA (Low-Income Population)**	-0.949	0.082	0.473	0.127	0.242	0.129	-0.23	0.104
**Lin BH, et al. (2011)**[49]**, USA (High-Income Population)**	-1.292	0.096	0.529	0.093	-0.054	0.13	-0.591	0.112
**Smith TA, et al. (2010)**[50]**, USA**	-1.264	0.089	0.557	0.095	0.222	0.126	-0.457	0.103
**Overall**	-1.299		0.388		0.129		-0.423	
**(LCI – HCI)**	(-1.089 - -1.509)		(0.0095 - 0.767)		(-0.085 - 0.342)		(-0.628 - -1.219)	

In all the next four cases the authors were contacted by email and could not provide any additional information to explain the missing values needed. To enable inclusion of the studies, we estimated the following values:

^1^Own-price elasticity: Consumer prices rose “by more than 3.4% on average” which led to a decrease in market share of 7.5%. Then -7.5/3.4 = -2.206. SE was inferred from Table VI: SE = (-0.07/1.16)*2.206 = 0.133.

^2^Information obtained from the paper: P < 0.05 at p(2-sides) = 0.05. Then SE = -[0.85]/1.96 = 0.434.

^3^The paper does not report SE, but only a p-value of 0.0000. It was used a website to derive an estimate of the Z-statistic (http://easycalculation.com/statistics/p-value-for-z-score.php). It was chose a value of 4.1, which is the lowest value that gives a two-sided p-value of 0.0000. Then SE = -[2.255]/4.1 = 0.550.

^4^SE inferred from mean and SE of grams of soft drink consumption (Table 4 in ref [48]): mean = -18.052, SE = 7.333, Ratio 7.33/-18.052 = -0.40622. Then SE = -4.445*-0.40622 = 1.806.

The numbers in parenthesis denote 95% confidence interval.

Table 3 also shows cross-price elasticities from the USA [49,50] and Mexico [44] Generally, the consumption of SSBs was compared with whole milk, fruit juices and diet soft drinks. The demand for these products was affected by SSBs prices. For fruit juices, the meta-analysis shows a cross-price elasticity of 0.388 (95% CI: 0.010 – 0.767), for whole milk a cross-price elasticity of 0.129 (95% CI: -0.085 – 0.342) and for diet soft drinks the cross-price elasticity is -0.423 (95% CI: -0.628 – -0.219). This means that fruit juices and perhaps whole milk act as substitutes for SSBs [44,45,52,53] and diet soft drinks are consumed in complement to SSBs [44,48,49] (See Table 1 for definitions).

Table 4 shows the impact on BMI, overweight and obesity due to a change in the price of different SSBs. It was not possible to perform a meta-analysis because of differences in interventions, outcomes and populations (e.g. households, individuals - adults, adolescents and children - as well as diverse range of food stores and vending machine outlets). The only available information was from the USA [12,23,47,50,52]. A range of estimates were reported, showing significant discrepancy between results. Han and Powell [52] show that for a 1% increase in SSB price, the point prevalence for obesity would reduce more in men (-0.34 percentage points) than in women (-0.05) [52]. Smith, et al. [50] found a reduction in the point-prevalence of overweight (-0.045 percentage points) and obesity (-0.03 percentage points) in adults [50]. Finkelstein, et al. [18] presents data at household level, for 20% increase in SSBs price the impact on BMI would be -0.065 kg/m2 [47]. The negative impact on BMI reported by Powell, et al. [23] is for specific taxes: the effect of a grocery soda tax was associated with a BMI increase of 0.012 kg/m2 and a tax on soft drinks sold via vending machines with a 0.011 kg/m2 increase. These effects were not statistically significant [23]. Fletcher, et al. [12] found a low level of impact in BMI (-0.0031 kg/m2) for adults and a non-significant impact (-0.015 kg/m2) in children and adolescents [12,29].

**Table 4:** The impact on obesity, overweight and BMI based in consumption change due to price increase of SSBs

**Author/year/country**	**Impact on BMI kg/m2**	**SE**	**Impact on overweight**	**SE**	**Impact on obesity prevalence**	**SE**	**Price increase (%)**
**Fletcher JM, et al. (2010a)**[12]**USA – Adults**	- 0.0031	0.0005	-0.0002	0.0001	-0.0001	-0.000	1%
**Fletcher JM, et al. (2010b)**[48]**USA – Children and adolescents**	- 0.015	0.016	-0.002	0.011	-0.009	-0.006	1%
**EA, et al. (2010)**[47]**USA**	- 0.065^1^	0.023					20%
**Han E, et al. (2011)**[52]**, USA – Women**					-0.05	0.287	10%
**Han E and Powell LM. (2011)**[52]**, USA – Men**					-0.34	0.381	10%
**Powell LM, et al. (2009)**[21]**USA – Vending Machine**	0.011	0.017					1%
**Powell LM, et al. (2009)**[23]**USA – Grocery shops**	0.0124	0.0124					1%
**Smith TA, et al. (2010)**[50]**, USA**			-0.045	N/R	-0.03	N/R	20%

^1^BMI calculation. Formula: Mass (kg)/(Height(m))^2 = 0.20 kg/1.76 m = **0.065**. Mass is 0.20 kg (Page 5. Table 3. 20%Tax on carbonated SSBs. All groups). Height: 1.76 (USA average height).

*N/R* Not reported.

## Discussion

This comprehensive literature review suggests that an increase in price of SSBs is associated with a decrease in consumption; and the higher the price increase, the greater the reduction in consumption. Also, as the price of SSBs rises, the consumption of fruit juices and whole milk tends to increase (although the evidence for the latter trend is not statistically significant), and the consumption of diet drinks decreases. The few available studies suggest that higher prices of SSBs may lead to modest reductions in weight in the population.

The intention of this study was to evaluate the available evidence worldwide. The pooled own-price elasticity was -1.30 (Table 3). These results are consistent with other meta-analyses including Powell et al. [18,37] which found an own-price elasticity of -1.21; and Andreyeva, et al. [38] which found an own-price elasticity of -0.79. Both of these studies used only USA data. There was insufficient evidence to undertake a separate meta-analysis for LMICs.

The possible reasons for the own-price elasticity outlier results [45,48] include that fact that one of the studies used data from France and the consumption patterns may differ from the USA. Context-specific evidence can however be important because different elasticities could be related to income or consumer preferences.

Several states in the USA have already implemented an excise tax on SSBs, but there is no experience from middle income countries where the SSB market is growing. Research from Mexico [44] and Brazil [46] is based on modelling a correlation between prices and consumption and provides useful information for middle income countries, since it can be used to assess the impact of taxes. Claro, et al. [46] presented two different own-price elasticities, for poor (-1.03) and non-poor (-0.63) (urban and rural area), which suggests that in Brazil, the poor are more price-sensitive than the more affluent [46].

The evidence from Brazil and Mexico is consistent with evidence from high income countries. This is important information for policy makers in other middle income countries, who can potentially draw on the pooled data until local evidence becomes available.

A surprising finding of the review was that the consumption of diet drinks may decrease as the price of SSBs increases [54]. Several explanations are possible but evidence is scarce. Andreyeva et al. suggest that bottlers and/or retailers could equalise prices between both types of beverages to counteract the tax [43] [REF]. People may consume diet soft drinks in the company of people who consume sweetened soft drinks, and if the price of the latter goes up, both might switch to alternative beverages. Media reports on the negative impact of SSBs that accompany new taxes may change cultural norms not only for SSBs but also for diet drinks, in a sort of 'contamination’ effect. Replacing SSBs with sugar-free beverages may not completely avoid health problems. Some studies suggest that the risk of developing metabolic syndrome increases by 34% with high consumption of diet soda [55,56]. In that light, a negative cross-price elasticity would be reassuring. However, although the results of our meta-analysis are statistically significant, the negative cross-price elasticity for diet drinks relies on only three studies, which is not a strong basis for conclusions.

Double-blind, randomised clinical trials in children and adolescents find that drinking sugar-free beverages rather than sugar containing beverages has the potential to significantly reduce weight gain and body fat gain [32,57]. There is good evidence of an association between SSB consumption and obesity. In addition, an increase in price of SSBs would likely reduce consumption [18,44]. This suggests that taxing SSBs effectively could result in reduced BMI, overweight and obesity among populations. Smith et al. [50] show a decrease in point prevalence for overweight (-0.045) and obesity (-0.03). The authors argue that a minor reduction in caloric intake will change the weight classification of these adults as most of them are marginally overweight or obese. Likewise, adults with a higher weekly or daily consumption would be affected more than those whose consumption is lower. In contrast, an increase in weight was found in the study by Powell, et al. [23] and a modest reduction in weight in the study by Fletcher, et al. [48], but each of these studies raises questions. The positive association between taxes and body weight that Powell, et al. [23] presented, may be related to the analysis focusing on specific taxes, i.e., a vending machine soda tax rate that appeared associated with a 0.011 kg/m2 increase in BMI. Such taxes may not be effective as people may purchase their SSBs via other outlets. Moreover, existing high levels of obesity should have prompted the imposition of taxes on all SSBs rather than on specific vending machine outlets. Fletcher et al. [48] provide some evidence that such a tax may reduce the weight among adolescents at risk of overweight, while there is no observed effect on those with normal weight. One study [48] notes that the impact may be minimal because of substitution with fruit juice and milk. The calories in these beverages could reduce the effect of price increases on SSBs; juice consumption as been associated with weight gain [53]. Even so, a switch to milk and fruit juice would still come with a health gain as these drinks contain calcium and vitamins.

Overall this review suggests that taxing SSBs may benefit health. Potential models for taxing SSBs can be drawn from the tobacco and alcohol excise tax experience. Because SSBs do not provide any nutritional value, and when consumed in excess can be harmful, it seems appropriate to consider a similar approach. This would mean that the tax is levied as a “specific tax” for example an “excise tax”, such as a sum per gram of added sugar, raising the net-of-tax price [45]. There are two important reasons for considering a tax on sugar content: Firstly, it would avoid substitution in favour of other products with high sugar content [45] and secondly, it might provide an incentive for industry to reduce the sugar content of SSBs. In contrast, if the tax is an “ad valorem” tax, the specific value of the tax will be based on the cost of the product – thus permitting a company to reduce the pre-tax price of their product, hence lowering the tax level and undermining its health impacts (albeit at the expense of profits). SSB price has been shown to have a dose–response relationship with consumption, with higher taxes resulting in greater reduction in SSB calories [45,58,59]. This consumption effect may be mitigated by consumers substituting products such as milk or fruit juice. An alternative option would be to tax all sugar when it leaves the factory or enters a country, using the argument that such a tax may be easier to implement and that sugar consumption in general is higher than is good for health [60]. Chriqui et al. [60] give a useful overview of SSB taxes applied in jurisdictions around the world.

Taxes generate revenue. For example, in the USA, soft drink revenue is approximately $70 billion per year, so a modest tax would generate billions of dollars [48]. The revenue from an excise tax could be used to support a variety of obesity reduction programmes, school nutrition programmes, or to finance a Health Promotion Foundation [61] that could advocate for healthy eating including further reductions in the consumption of SSBs. A tax might also be used to subsidise alternative healthy drinks to reduce their price and thus encourage consumption [62,63]. In areas where people drink SSBs because they do not have access to clean water, ensuring universal access to clean, piped water should be a priority.

One argument against the imposition of an excise tax alone is that it is regressive [12,49,64]. Lower-income households tend to spend a greater portion of their income on consumable goods than higher-income households. Relative to income, a SSB tax would therefore affect low-income people more than high-income people. However, low income households, as a group, are also likely to reap greater benefit. To the extent that low-income individuals are more price sensitive, they will be more likely to cut back on the intake of taxed SSBs, often from a higher consumption level and with a higher BMI, and thus experience greater health gain [64]. This gives ground to consider a simultaneous subsidy of healthy foods such as fruit and vegetables. In the past, those most susceptible to obesity and cardiovascular diseases were among the wealthier in the population but this is no longer the case. Low income earners are now a population with high consumption of unhealthy obesogenic food [65,66]. Also in many LMICs, the prevalence of obesity is growing more rapidly in low socioeconomic groups [67]. Upcoming research should estimate price elasticities in low- and middle-income countries and identify potential health gains from taxes combined with subsidies of healthy food.

As far as we know, this is the first global overview of the effect of SSB price on consumption and body weight and, in the absence of good quality local evidence, may inform policy decisions worldwide. The limitations are twofold. First, in LMICs, consumption patterns and price sensitivities may differ from HIC (although the evidence from Mexico and Brazil does not support this). Secondly, the data included in the meta-analysis are from heterogeneous populations with various data sources involving households, individuals (adults, adolescents and children) as well as a diverse range of food stores and vending machine outlets.

## Conclusions

An increase in price of SSBs is associated with a decrease in consumption; and the higher the price increase, the greater the reduction in consumption. Also, as the price of SSBs rises, the consumption of fruit juices and whole milk tends to increase and the consumption of diet drinks decreases. The alternative beverages are most likely better for health than SSBs. The few available studies suggest that higher prices of SSBs may lead to modest reductions in weight in the population. This evidence and the link between obesity and SSBs and its health outcomes should be sufficient for policy makers to consider SSB taxation as part of a package of intervention designed to reduce the health and economic burden due to obesity. Future research should address the consequences of a tax on SSBs, including the health gains, population affected and the impact on the macroeconomic environment including jobs, monetary savings to the health sector, implementation costs and revenue generated for the government [68]. Full cost-effectiveness studies would provide stronger evidence and allow policy makers to weigh these factors. To enhance relevance for any particular jurisdiction, such studies should use data specific to the countries or sub-regions, and be undertaken with sound understanding of context-specific policies, history and socio-cultural preferences.

## Abbreviations

SSBs: Sugar sweetened beverages; LMICs: Low and middle income countries; NCDs: Non-communicable diseases; HICs: High income countries; GBD: The Global Burden of Disease; NBER: National Bureau of Economics Research; RePEc: Research Papers in Economics.

## Competing interests

The authors have declared that no competing interests exist. The content of the work presented here is solely the responsibility of the authors and does not necessarily represent the official views of the IDRC.

## Authors’ contributions

MC: Performed the literature review and prepared the first draft of the manuscript. LV: Supervised the meta-analysis and provided comments on all drafts of the manuscript. ST: Provided comments on the manuscript and critically revised it for important intellectual content. MB: Contributed to the conception of the study, supervised the initial literature review and provided critical comments on several draft of the manuscript. KH: Made substantial contributions to the conception of the study, provided critical comments to all drafts. All authors have given final approval of the manuscript to be submitted.

## Pre-publication history

The pre-publication history for this paper can be accessed here:

http://www.biomedcentral.com/1471-2458/13/1072/prepub
